# Percutaneous Management of Pyogenic Liver Abscesses

**DOI:** 10.1155/1989/87603

**Published:** 1989

**Authors:** O. Helenon, M. Krachte, D. Mathieu, N. Vasile, N. Rotman, P. –L. Fagniez

**Affiliations:** 1 Department of Radiology Henri Mondor Hospital Créteil France; 2 Department of General Surgery Henri Mondor Hospital Créteil France; 3 Dep. of General Surgery Henri Mondor Hospital 51, Ave du Mal de Lattre de Tassigny Créteil 94010 France

## Abstract

Twenty-four pyogenic liver abscesses have been treated during a six-year period percutaneously. Percutaneous management included percutaneous drainage and fine needle aspiration under ultrasound or CT scan guidance. Percutaneous management was successful in 92% of cases, and no further treatment was required in 91% of these.

One patient died, giving a mortality rate of 4.1%. There were no complications related to this method. The authors conclude that percutaneous management of pyogenic liver abscesses should be attempted in all cases, since results compare favourably with surgical procedures.


*HPB Surgery*, 1989, Vol. 1, pp. 329–335
Reprints available directly from the publisher
Photocopying permitted by license only
© 1989 Harwood Academic Publishers GmbH
Printed in Great Britain

### PERCUTANEOUS MANAGEMENT OF PYOGENIC LIVER ABSCESSES*

O. HELENON[1], M. KRACHT[2], D. MATHIEU[1], N. VASILE[1], N. ROTMAN[2], P.-L. FAGNIEZ**[3]

*[1]Department of Radiology, [2]Department of General Surgery, Henri Mondor Hospital, Créteil, France*

*(Received 7 November, 1988)*

Twenty-four pyogenic liver abscesses have been treated during a six-year period percutaneously. Percutaneous management included percutaneous drainage and fine needle aspiration under ultrasound or CT scan guidance. Percutaneous management was successful in 92% of cases, and no further treatment was required in 91% of these.

One patient died, giving a mortality rate of 4.1%. There were no complications related to this method. The authors conclude that percutaneous management of pyogenic liver abscesses should be attempted in all cases, since results compare favourably with surgical procedures.

KEY WORDS: Liver, pyogenic abscess, percutaneous management

### INTRODUCTION

Percutaneous management of intraabdominal abscesses using fine needle aspiration and catheter drainage has become an alternative to surgical management. Several reports have emphasized its value in the management of pyogenic liver abscesses.

Fine needle aspiration, in addition to its diagnostic value, allows specific antibiotherapy and complete evacuation of the abscess cavity, which should facilitate the efficiency of antibiotics[1]. Percutaneous drainage represents an alternative to surgery and is advocated because of its high success rate[1–6].

This study reports our experience with percutaneous management of pyogenic liver abscesses during the past six years.

### METHODS

Between January 1982 and December 1987, 24 patients with pyogenic liver abscesses were treated by percutaneous fine needle aspiration and/or percutaneous drainage under ultrasound or CT scan guidance. There were 18 men and 6 women with a mean age of 53 years (range 14–82 years).

Percutaneous drainage was used in large abscesses to ensure a rapid evacuation of

---

* This paper has been presented in poster form on the 2nd World Congress on HPB Surgery, Amsterdam 1988.

** Address correspondence to: P.-L. Fagniez, Dpt. of General Surgery, Henri Mondor Hospital, 51, Ave du Mal de Lattre de Tassigny, 94010 Créteil, France the residual cavity. Fine needle aspiration with an 18–21 Gauge Chiba needle was used in the case of small abscesses (less than 4 cm in diameter), or in the case of abscesses very close to the inferior vena cava. A combined approach was mainly chosen in the case of multiple abscesses. The drainage procedure, preceded by a diagnostic fine needle aspiration, consisted in the introduction under CT scan guidance of 8 to 12 French multiperforated single lumen pigtail catheters. In one case of a very large abscess the catheter was introduced under ultrasound guidance.

The percutaneous drains were flushed twice daily with 10 to 15 ml of a sterile saline solution to ensure their permeability.

Antibiotherapy was given to all patients, beginning usually with broadspectrum antibiotics such as cephalosporins, which were changed, if necessary, according to pus isolated organism sensitivities. The catheter was removed when the clinical and radiological course was satisfactory, i.e. when there was no further pyrexia, normal white cell count and disappearance of the abscess cavity on follow-up sonogram or CT scans. The mean duration of drainage was 12 days (range 7–21 days).

Etiological factors are listed in Table 1. Cryptogenic abscess was the most frequent condition, followed by abscesses of biliary or portal origin. In all cases with clinical suspicion, ultrasound and/or CT assessment of the hepatic lesions lead to percutaneous management. Of the 24 patients, 6 were treated by percutaneous fine needle aspiration alone (Figure 1), while 16 were treated by percutaneous drainage, which was associated to fine needle aspiration in 6 cases (Figure 2 Table 2). Percutaneous management was unsuccessful in two patients.

**Table 1** Etiological factors of pyogenic liver abscess

| | |
|---|---|
| Cryptogenetic | 8 |
| Diverticulitis | 4 |
| Pyogenic cholangitis | 3 |
| Acute cholecystitis | 2 |
| Dental abscess | 2 |
| Crohn's disease | 1 |
| Ulcerative colitis | 1 |
| Mesenteric necrosis | 1 |
| Renal abscess | 1 |
| Rendu-Osler's disease | 1 |

The average hospital stay was 6 weeks (range 3 weeks to 4 months), largely due to the fact that the initial cause of the abscess was treated during the same hospital stay.

### RESULTS

Percutaneous management was successful in 22 out of 24 patients (92%) without complications related to this method. In two patients in our early experience, percutaneous aspiration failed under ultrasound guidance. In both cases, abscesses were relatively small (4 and 3 cm diameter) and located in the dome of the liver. One patient came to surgery, while the other recovered without further treatment under antibiotic treatment. We have not observed such difficulties since 1983.

In six cases, percutaneous fine needle aspiration with complete evacuation of the abscess cavity was sufficient. In 16 cases, percutaneous drainage was required and was associated with fine needle aspiration in six.

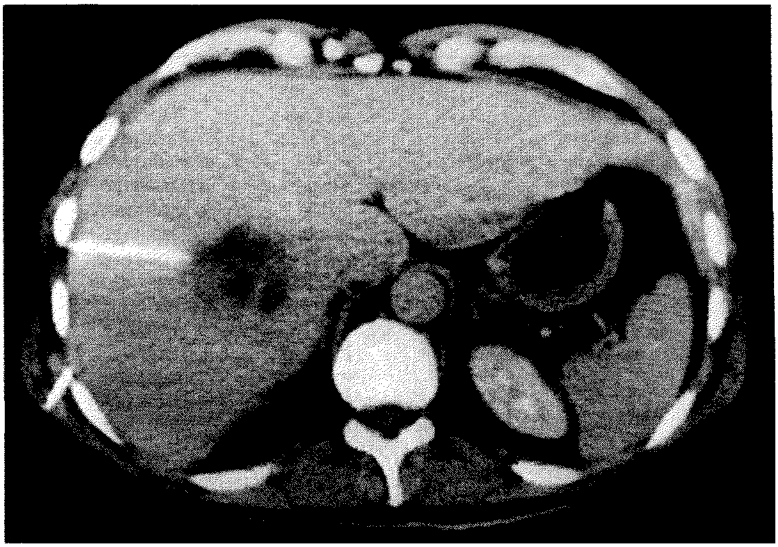

**Figure 1** Percutaneous aspiration using an 18 gauge needle of a multiloculated hypodense abscess of less than 4 cm in diameter.

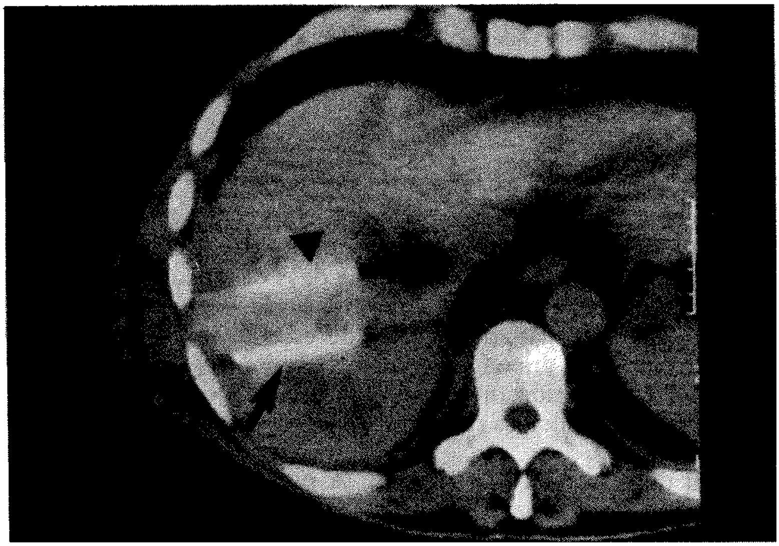

**Figure 2A** Percutaneous management of multiple abscesses of the right lobe of the liver. Double approach with percutaneous drainage (arrow) of the larger abscess, associated with fine needle aspiration of a small abscess close to the IVC (arrowhead).

**Table 2** Clinical and Bacteriological Data

| *Case* | *Sex* | *Age* | *Abscess* | *Blood* | *Pus* | *Percutaneous* | *Outcome* |
|---|---|---|---|---|---|---|---|
| 1 | M | 29 | Single | – | Strepto | Drainage | Recovery |
| 2 | F | 67 | Multiple | – | E. Coli | Aspiration | Recovery |
| 3 | M | 30 | Multiple | – | – | Failure | Surgery |
| 4 | M | 32 | Multiple | Strepto | – | Failure | Recovery |
| 5 | F | 64 | Multiple | – | Strepto | Drainage+ aspiration | Recovery |
| 6 | M | 80 | Multiple | E. Coli | Bacteroides | Drainage+ aspiration | Recovery |
| 7 | F | 80 | Multiple | – | Strepto | Aspiration | Recovery |
| 8 | M | 71 | Multiple | E. Coli | E. Coli | Drainage | Surgery |
| 9 | M | 27 | Single | – | – | Drainage | Recovery |
| 10 | M | 27 | Single | Strepto | Proteus | Drainage | Recovery |
| 11 | M | 29 | Multiple | – | – | Aspiration | Recovery |
| 12 | F | 39 | Single | – | Citrobact | Drainage | Recovery |
| 13 | M | 82 | Single | Bacteroides | Bacteroides | Drainage | Recovery |
| 14 | F | 84 | Multiple | E. Coli Strepto | Proteus | Drainage + aspiration | Died |
| 15 | F | 54 | Single | Proteus Bacteroides Strepto | Stepto. Mill Strepto Bovis | Aspiration | Recovery |
| 16 | M | 77 | Single | – | – | Aspiration | Recovery |
| 17 | M | 27 | Single | – | Strepto. Mill Hemophilus | Drainage | Recovery |
| 18 | M | 16 | Single | – | – | Drainage | Recovery |
| 19 | M | 28 | Single | Fusobact. | Fusobact. | Drainage | Recovery |
| 20 | M | 27 | Single | – | – | Drainage | Recovery |
| 21 | M | 32 | Multiple | – | Staph. aur. | Drainage+ aspiration | Recovery |
| 22 | M | 52 | Multiple | Strepto. | Strepto. | Drainage+ aspiration | Recovery |
| 23 | M | 52 | Multiple | – | Strepto. | Drainage+ aspiration | Recovery |
| 24 | M | 14 | Single | – | Staph. Aur. | Aspiration | Recovery |

Percutaneous management failed in two cases in spite of adequate and functional drainage; one patient had an abscess close to a perforated gangrenous cholecystitis, and persistent sepsis indicated surgery after six days of percutaneous drainage; the other patient had multiple abscesses secondary to an obstructed Roux-en-Y hepatico-jejunostomy. This patient was considered unfit for surgical treatment and died in spite of successful drainage of overwhelming sepsis and multiple organ failure, giving an overall mortality rate of 4.1%. All other patients recovered uneventfully. This gives a success rate of nearly 91%, provided the abscesses were attained percutaneously.

Bacteriological data are listed in Table 2. A polymicrobic abscess content was observed in five cases (20.8%).

Blood cultures were positive in only 9 cases, whereas in 17 cases organisms were isolated from the pus obtained during the percutaneous aspiration. In twelve cases, aspiration allowed germs to be demonstrated which were not found in blood cultures. In seven cases, the pus obtained remained sterile. In all these cases, broad spectrum antibiotherapy had been started before the diagnosis was established on the grounds of persistent fever.

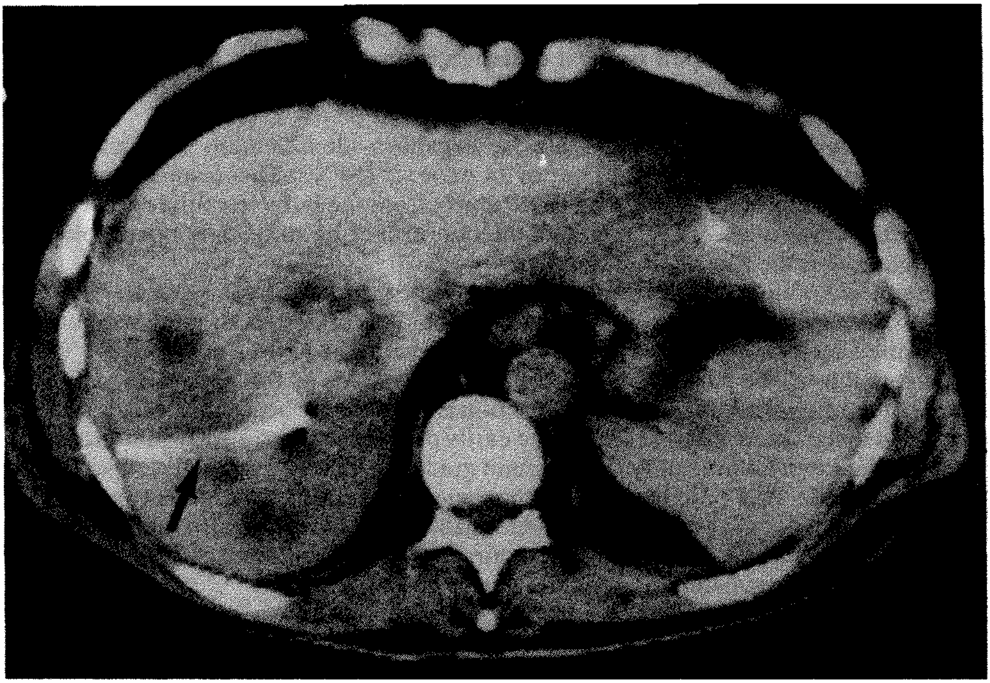

**Figure 2B** Percutaneous management of multiple abscesses of the right lobe of the liver. The aspirated cavity near the IVC has collapsed. The drain is kept in place for one week (arrow).

### DISCUSSION

Percutaneous management of pyogenic liver abscess gives a mean success rate of about 80%[1, 4-6]. Gerzof, in his review of the literature, found that the overall mortality of percutaneous management was lower (3 to 4%) than with surgical procedures[1]. In our series of 20 surgically treated pyogenic liver abscesses between 1970 and 1980, we experienced a mortality rate of 30%[7]. Recent series give even better results of percutaneous management with a mortality rate between 0 and 2.5%[1,3,4,6] except for Bertel et al. who observed a 13% mortality[5]. This rate corresponded to patients with necrotic, superinfected hepatic tumours and concomitant carcinomatosis.

Thus most authors advocate initial percutaneous drainage combined with antibiotic treatment in the management of pyogenic liver abscesses[1,2,4,6,8–12]. Furthermore, several authors have reported a successful outcome after short time drainage (27–72 hours)[4] and after simple aspiration combined with germ-specific antibiotherapy which might be sufficient in some cases[13,14].

Failure may be due to the high viscosity of the abscess material[4,5], which may be encountered in case of superinfected hematoma or necrotic tumors. Although preoperative imaging indicated slough in several patients, this was never a cause of failure, since daily lavage seems an adequate treatment via a percutaneous drain.

In our experience, percutaneous management failed to obtain recovery in two cases which were both of biliary origin. Although both cases were successfully drained, a surgical procedure was required but could only be performed in one. This does not, in our opinion, diminish the value of percutaneous management since in both cases we obtained early bacteriological data which permitted adequate antibiotic treatment. Open surgical drainage is unsuccessful or incomplete[1,4]. The necessity of surgical management of the underlying disease does not necessarily preclude percutaneous management.

Bacteriological data show that precise organism identification was possible by percutaneous management in 13 out of 24 patients (54%). This results from a high rate of negative blood cultures and particularly from the polymicrobial nature of the abscesses in five cases. This highlights the importance of immediate bacteriological examination of the aspirated material.

The choice of the percutaneous approach should be guided by the size, the location of the abscess and the viscosity of its content. Percutaneous drainage should be performed for abscesses greater than 4 cm in diameter and of easy access. When transgression of the parietal pleura cannot be avoided, as for the abscesses of the dome of the right lobe, fine needle aspiration is the safest procedure[15]. Furthermore, we believe that these abscesses are more easily drained under CT scan guidance since we observed two failures under ultrasound guidance in our early experience.

We conclude that percutaneous management is a safe and efficient method of treatment of pyogenic liver abscesses, diminishing the mortality rate from 30 to 4.1% in our experience. Surgery may be required for the treatment of the underlying disease or in case of failure of percutaneous management.

### *References*

1. Gerzof S.G., Johnson W.C., Robbins A.H., and Nabseth D.C. (1985) Intrahepatic pyogenic abscesses: treatment by percutaneous drainage. *American Journal of Surgery,* **149,** 487–492
2. Mandel S.R., Boyd D., Jacques P.F., Mandell V., and Staab E.V. (1983) Drainage of hepatic, intraabdominal, and mediastinal abscesses guided by computerized axial tomography. Successful alternative to open drainage. *American Journal of Surgery,* **145,** 120–124
3. Bernardino M.E., Berkman W.A., Plemmons M., Sones P.J.Jr, Price R.B., and Casarella W.J. (1984) Percutaneous drainage of multiseptated hepatic abscess. *Journal of Computerized Assisted Tomography,* **8,** 38–41
4. Johnson R.D., Mueller P.R., Ferrucci J.T.Jr, Dawson S.L., Butch R.J., Papanicolaou N., Van sonnenberg E., Simeone J.F., Wittenberg J. (1985) Percutaneous drainage of pyogenic liver abscesses. *American Journal of Radiology,* **144,** 463–467
5. Bertel C.K., Van Heerden J.A., and Sheedy P.F. (1986) Treatment of pyogenic hepatic abscesses. Surgical vs. percutaneous drainage. *Archives of Surgery,* **121,** 554–558
6. Gyorffy E.J., Frey C.F., Silva J. Jr, and McGahan J. (1987) Pyogenic liver abscess. Diagnostic and therapeutic strategies. *Annals of Surgery* **206,** 699–705
7. Fagniez P.L., Villet R., Thomsen C., Hannoun S., Julien M., and Germain A. (1982) Abcès du foie à pyogènes: une étude de vingt cas opérés. *Chirurgie* **108,** 37–42
8. Sheinfeld A.M., Steiner A.E., Rivkin L.B., Dermer R.H., Shemesh O.N., and Dolberg M.S. (1982) Transcutaneous drainage of abscesses of the liver guided by computed tomography scan. *Surgery, Gynecology & Obstetrics* **155,** 662–666
9. Kraulis J.E., Bird B.L., and Colapinto N.D. (1980) Percutaneous catheter drainage of liver abscess: an alternative to open drainage? *British Journal of Surgery* **67,** 400–402
10. Greenwood L.H., Collins T.L., and Yrizarry J.M. (1982) Percutaneous management of multiple liver abscesses. *American Journal of Radiology* **139,** 390–392
11. Northover J.M.A., Jones B.J.M., Dawson J.L., and Williams R. (1982) Difficulties in the diagnosis and management of pyogenic liver abscess. *British Journal of Surgery* **69,** 48–51
12. Haaga J.R. and Weinstein A.J. (1980) CT guided percutaneous aspiration and drainage of abscesses. *American Journal of Radiology* **135,** 1187–1194
13. McFadzean A.J.S., Chang K.P.S., and Wong C.C. (1953) Solitary pyogenic abscess of the liver treated by closed aspiration and antibiotics. A report of 14 consecutive cases with recovery. *British Journal of Surgery* **41,** 141–152
14. Dietrick R.B. (1984) Experience with liver abscess. American Journal of Surgery **147,** 288–291
15. Neff C.C., Mueller P.R., Ferrucci J.T. Jr, Dawson S.L., Wittenberg J., Simeone J.F., Butch R.J. and

Papanicolao N. (1984) Serious complication following transgression of the pleural space in drainage procedures. *Radiology* **152,** 335–341

*Accepted by L. Blumgart on 27 November 1988.*

